# Developing a Semi-Quantitative Occupational Risk Prediction Model for Chemical Exposures and Its Application to a National Chemical Exposure Databank

**DOI:** 10.3390/ijerph10083157

**Published:** 2013-07-25

**Authors:** Shih-Min Wang, Trong-Neng Wu, Yow-Jer Juang, Yu-Tung Dai, Perng-Jy Tsai, Chiu-Ying Chen

**Affiliations:** 1 Department of Occupational Safety and Health, College of Public Health, China Medical University, 91 Hsueh-Shih Road, Taichung 40402, Taiwan; E-Mail: smwang626@gmail.com; 2 Graduate Institute of Biostatistics, China Medical University, 91 Hsueh-Shih Road, Taichung 40402, Taiwan; E-Mail: tnwu@mail.cmu.edu.tw; 3 Department of Occupational Safety and Health, Chung Hwa University of Medical Technology, 89, Wenhwa 1st St., Rende District, Tainan 71701, Taiwan; E-Mail: Jyjstone@mail.hwai.edu.tw; 4 Department of Occupational Safety and Health, Chang Jung Christian University, 1 Changda Rd., Gueiren District, Tainan 71101, Taiwan; E-Mail: ytdai@mail.cjcu.edu.tw; 5 Department of Environmental and Occupational Health, Medical College, National Cheng Kung University, 138 Sheng-Li Road, Tainan 70403, Taiwan; 6 Department of Public Health, College of Public Health, China Medical University, 91 Hsueh-Shih Road, Taichung 40402, Taiwan

**Keywords:** semi-quantitative risk predicting model, chemical exposure, exposure databank, control strategy

## Abstract

In this study, a semi-quantitative occupational chemical exposure risk prediction model, based on the calculation of exposure hazard indexes, was proposed, corrected, and applied to a national chemical exposure databank. The model comprises one factor used to describe toxicity (*i.e.*, the toxicity index), and two factors used to reflect the exposure potential (*i.e.*, the exposure index and protection deficiency index) of workers exposed to chemicals. An expert system was used to correct the above proposed model. By applying the corrected model to data obtained from a national occupational chemical hazard survey program, chemical exposure risks of various manufacturing industries were determined and a national control strategy for the abatement of occupational chemical exposures was proposed. The results of the present study would provide useful information for governmental agencies to allocate their limited resources effectively for reducing chemical exposures of workers.

## 1. Introduction

Assessing occupational chemical exposure risks is important for enacting appropriate exposure abatement strategies for protecting the health of workers. To date, such assessments have been conducted mostly by using the quantitative exposure monitoring technique [1,2,3,4]. However, difficulties usually arise with industries mainly due to the more hazardous chemicals used in some industries, raising costs and the need for more intensive manpower for conducting exposure assessments. An alternative strategy, known as the control banding (CB) strategy, has recently been developed for risk assessment and management of chemical exposure hazards in workplaces [5,6,7]. The CB strategy was developed initially by the United Kingdom (UK) Health and Safety Executive (HSE) in the late 1980s, and was known as the Control of Substances Hazardous to Health Essentials (COSHH Essentials) [8,9]. Subsequently, the International Labor Organization (ILO) developed a Chemicals Control Tool Kit (CCTK) suitable for industries of different countries [10]. The conceptual basis for both COSHH Essentials and CCTK is to group chemical exposure risks into “bands” based on potential health hazards (*i.e.*, the hazard bands), and exposure potentials (*i.e.*, exposure bands). For COSHH Essentials, only the Risk-phrases (R-phrases) are adopted for determining the hazard bands of chemicals. But for CCTK, either R-phrases or hazardous categories assigned by the Globally Harmonized System for the Classification and Labeling of Chemicals (GHS) are used in determining the hazard bands of the chemicals involved. To determine the exposure band of a chemical, its quantity in use together with its volatility (for liquid) or dustiness (for solid) are considered in both COSHH Essentials and CCTK [8,11,12,13]. Based on the same principle, many other CB strategies, such as the two-stage risk assessment strategy (Rogetox) developed in Belgium and the semi-quantitative risk assessment (SQRA) developed in Singapore, have been developed and used in different countries recently [6,14].

In principle, the development of a CB strategy is not intended to replace the quantitative monitoring approach, but rather is used as a screening and handy tool for small-and medium-size enterprises (SMEs) when conveniently prioritizing exposure risks imposed on workers [15,16,17]. Therefore, the number of factors contained in a CB should be limited to reduce its complexity and increase its applicability for people with different backgrounds [18]. However, oversimplifying the number of factors might result in decreasing the effectiveness of a CB when determining chemical exposure risks. For example, if health hazard potentials are determined only based on the R-phases of the involved chemicals, the health hazard potentials could not be further discriminated even they represent very different occupational exposure limits but with the same R-phrase. Theoretically, the use of effective engineering control devices or personal protective equipment (PPE) would affect the exposure potential. Therefore, considering only the quantity of the chemical in use and its volatility (or dustiness) are obviously inadequate to assess its intrinsic exposure potential. Moreover, inconsistencies have been found between CB-predicted exposure risks and environmental monitoring results, even in SMEs [19,20,21]. Hashimoto *et al.* found the predicted risk level obtained from COSHH Essentials tends to provide a safe-sided (over-controlling) judgment [19]. Tischer *et al.* found that the level of agreement between the measurement data and the CB predicted ranges is only reasonably good for solid-phase substances (powders, dusts). With a large amount of liquid-phase chemicals used in industries, a study found CB predicted risk levels higher than those classified according to the monitoring data, yet, an opposite effect was reported with a small amount of liquid-phase chemicals used in industries [21]. In sum, both COSHH Essentials and CCTK seem inadequate to assess exposure risk levels of workers for industries with complicated manufacturing processes and exposure scenarios.

In Taiwan, a national-wide occupational chemical exposure survey program was conducted during the period from 2006 to 2009 by governmental industrial hygiene inspectors. Because of their expertise in industrial hygiene, chemicals used in the surveyed industry were identified and their quantities were carefully recorded. In addition, administrative and engineering control measures adopted by the industry were also identified and recorded. In this study, a more sophisticated semi-quantitative chemical exposure risk predicting model, based on the concept of CB, was proposed. The developed model was corrected using the expert system (ES). Then, the exposure risk levels for various industries were determined using the corrected predicting model. Finally, the above results were used as a basis for initiating a suitable national chemical exposure abatement strategy.

## 2. Material and Methods

### 2.1. Developing a Chemical Exposure Risk Predicting Model

In this study, a semi-quantitative occupational chemical exposure risk predicting model, named Exposure Hazard Index (EHI), was proposed. Based on the CB strategy concept, the toxicity and exposure potential of chemicals were two factors contained in the framework of the developed EHI model. The toxicity of a given chemical was rated based on its toxicity index (TI). For exposure potential, two indices were considered, including the exposure index (EI) and protection deficiency index (PDI). The EHI proposed in this study is described as below:

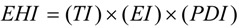
(1)


#### 2.1.1. Proposed Toxicity Index (TI)

In order to easily assess the toxicity of a given chemical, its time-weighted-average occupational exposure limit (OEL-TWA) was used as the parameter for determining its toxicity index (TI). Considering the toxic effect associated with a chemical exposure could be proportional to the logarithm of the exposure concentration of the given chemical, the above concept had been adopted for categorizing the toxicities of various involved chemicals [22,23]. By using the same concept, we assumed a chemical A has an OEL-TWA ten times higher than that of chemical B, then the TI for chemical A would be only one half the size as that of chemical B. As a result, Table 1 shows the eight TI levels proposed by the present study according to the ranges of OEL-TWA of chemicals. Considering those chemicals with a carcinogenic effect and some new chemicals do not have an OEL-TWA, their TI values were assigned pragmatically the same as the chemical with the lowest OEL-TWA value (*i.e.*, 0.001 ppm or TI = 128). For a chemical that only has an OEL-Ceiling, its OEL-TWA was assigned pragmatically as three times the size as that of OEL-Ceiling.

**Table 1:** Originally proposed ratings for toxicity index (TI), exposure index (EI), management index (MI) and protection index (PI).

Index	Rating
**TI ** (rated based on OEL-TWA)	
OEL-TWA > 1,000 ppm	1
1,000 ppm ≥ OEL-TWA > 100 ppm	2
100 ppm ≥ OEL-TWA > 10 ppm	4
10 ppm ≥ OEL-TWA > 1 ppm	8
1 ppm ≥ OEL-TWA > 0.1 ppm	16
0.1 ppm ≥ OEL-TWA > 0.01 ppm	32
0.01 ppm ≥ OEL-TWA > 0.001 ppm	64
0.001 ppm ≥ OEL-TWA	128
**EI **(rated based on exposure duration; ED)	
ED < 2 h	0.30
2 ≤ ED < 4 h	0.60
ED ≥ 4 h	1.00
**MI** (rated based on the number of implemented management measures; N)	
N = 0	0.00
N = 1	0.25
N = 2	0.50
N = 3	0.75
N = 4	1.00
**PI **(rated based on the combination of implemented control measures)	
EEn+ PPEn	0.00
EEn+ PPEe	0.20
EEp+ PPEn	0.30
EEp+ PPEe	0.50
EEe+ PPEn	0.80
EEe+ PPEe	1.00

#### 2.1.2. Proposed Exposure Index (EI)

In the present study, the EI for a chemical exposure was determined according to the exposure duration of workers for the chemical of interest. We categorized the exposure duration into three categories: less than 2 h, from 2 h to less than 4 h, and equals to or more than 4 h with ratings for EI as 0.3, 0.6, and 1.0, respectively (Table 1).

#### 2.1.3. Proposed Protection Deficiency Index (PDI)

The PDI index comprises two factors: the management index (MI) and protection index (PI). The MI rating was based on the number of management measures implemented in the workplace. Four management measures were considered for rating MI: the availability of safety and health personnel, material safety data sheet (MSDS), standard operating procedures (SOP) and training programs for handling hazardous materials. Table 1 shows the proposed MI ratings based on the number of management measures implemented in the workplace concerned. Here, MI was rated as 0, 0.25, 0.50, 0.75, and 1.00 when 0, 1, 2, 3, and 4 management measures were implemented in the workplace, respectively (Table 1).

As PI represents the effectiveness of control strategies adopted in the involved workplace, we then considered the effectiveness of both engineering controls and administrative controls for its rating. For engineering controls, both enclosure and local exhaust ventilation were considered as effective engineering control measures (EEe), but general ventilation was considered as a partially effective engineering control measure (EEp). Those not implementing any of the above engineering control measures in the workplace were denoted as EEn. Regarding the administrative control, we only considered the use of personal protective equipment (PPE). Workplaces providing workers with appropriate PPE or not were designated PPEe and PPEn, respectively. Table 1 depicts the PI ratings based on the combination of the engineering controls (*i.e.*, EEe, EEp and EEn) and administrative control measures (*i.e.*, PPEe and PPEn) adopted by the industry. Table 1 shows PI rated as 1.00, 0.80, 0.50, 0.30, 0.20 and 0.00 for EEe + PPEe, EEe + PPEn, EEp + PPEe, EEp + PPEn, EEn + PPEe and EEn + PPEn, respectively. In principle, higher PI and MI values indicate lower protection deficiency index (PDI), suggesting lower exposure probability of workers exists, therefore, the following expression is proposed to calculate the PDI:

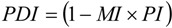
(2)

### 2.2. Correcting the Developed Exposure Hazard Index (EHI)

The major challenge for developing a semi-quantitative chemical exposure risk predicting model is how to choose and rate parameters appropriately. As expert systems have been widely adopted for rating workers’ exposures [24,25], we used one in the present study for correcting the proposed EHI prediction model. In principle, the expert system could allocate the occupational exposure by the well trained raters according to the detailed information about hazardous substances, workplace conditions and exposure factors. In this study, 20 governmental industrial hygiene inspectors, each with at least a 15-years of field experience, were chosen as the members of the expert system.

Information on the toxicity of involved chemicals, exposure durations, management measures and control measures implemented in the involved workplaces were collected from seven enterprises by three senior industrial hygiene inspectors. A total of nine chemicals were used in all selected enterprises. In Taiwan, the OEL-TWAs of these chemicals ranges from 0.02 ppm (MDI) to 750 ppm (acetone). Workers’ exposure durations ranged from 10 min to 250 min. The number of management measures implemented in workplaces ranged from one to four measures. For engineering controls, three, two, and two enterprises provided effective engineering control measures (EEe), partially effective engineering control measures (EEp), and no engineering control measures (EEn), respectively. On the other hand, only three enterprises provided workers with suitable PPE. Table 2 lists the results obtained from the above survey. The above information was sent to each of 20 members in the expert system to rate TI, EI, MI and PI based on their professional judgment.

**Table 2:** The survey results obtained from the seven selected enterprises, and the rating results for toxicity index (TI), exposure index (EI), and protection index (PI) obtained from the originally proposed ratings (TI_ori_, EI_ori_, and PI_ori_), expert system (TI_E__S_, EI_E__S_, and PI_E__S_), and after being corrected (TI_cor_, EI_cor_, and PI_cor_).

Survey results obtained from the 7 selected enterprises	Ratings of indices		
**Compounds and their OEL-TWAs**	**TI_ori_**	**TI_E__S_**	**TI_cor_**
Toluene diisocyanate, TDI (0.005 ppm)	64.00	86.31 ± 6.28	88.10
Methylene bisphenyl isocyanate, MDI (0.02 ppm)	32.00	56.71 ± 4.34	58.04
N, N-Dimethylformamide, DMF (10 ppm)	8.00	10.23 ± 1.03	8.94
Ethylene glycol, EG (50 ppm)	4.00	4.52 ± 0.67	5.51
Methylene chloride, MC (50 ppm)	4.00	4.52 ± 0.67	5.51
Toluene, Tol (100 ppm)	4.00	3.86 ± 0.37	4.47
Methyl ethyl ketone, MEK (200 ppm)	2.00	3.09 ± 0.42	3.63
Ethyl acetate, EAc (400 ppm)	2.00	2.91 ± 0.42	2.94
Acetone (750 ppm)	2.00	2.30 ± 0.36	2.44
**Workers’ exposure durations (EDs) **	**EI_ori_**	**EI_E__S_**	**EI_cor_**
10 min	0.30	0.02 ± 0.01	0.02
30 min	0.30	0.07 ± 0.02	0.07
40 min	0.30	0.09 ± 0.05	0.10
48 min	0.30	0.12 ± 0.05	0.11
75 min	0.30	0.14 ± 0.08	0.18
225 min	0.60	0.53 ± 0.11	0.52
250 min	1.00	0.60 ± 0.13	0.58
**Survey results obtained from the 7 selected enterprises**	**Ratings of indices**		
**Numbers of implemented management measures (N)**	**MI_ori_**	**MI_E__S_**	**MI_cor_**
0	0.00	0.00 ± 0.00	0.18
1	0.25	0.38 ± 0.36	0.39
2	0.50	0.59 ± 0.06	0.60
3	0.75	0.82 ± 0.06	0.81
4	1.00	1.00 ± 0.00	1.00
**The combination of implemented control measures**	**PI_ori_**	**PI_E__S_**	**PI_cor_**
EEn + PPEn	0.00	0.00 ± 0.00	0.00
EEn + PPEe	0.20	0.18 ± 0.06	0.18
EEp + PPEn	0.30	0.42 ± 0.03	0.42
EEp + PPEe	0.50	0.61 ± 0.12	0.61
EEe + PPEn	0.80	0.74 ± 0.04	0.74
EEe + PPEe	1.00	1.00 ± 0.00	1.00

Ratings obtained from the expert system (mean ± standard deviation) were compared with those originally proposed to examine the consistency between both rating systems. If consistency could be obtained, the results obtained from the expert system would serve as the basis for correcting the originally proposed model.

### 2.3. Application to a National Chemical Hazard Survey Databank

A national occupational chemical hazard survey program was conducted from 2006 to 2009 by all local governmental labor inspector offices in 25 manufacturing industries. The selection of enterprises for each manufacturing industry was based on a random sampling strategy, with the number of selected enterprises proportional to the number of enterprises in a given industry. A total of 702 enterprises were chosen. During the survey, the basic information about the enterprise (the type of the industry, manufacturing products, manufacturing processes, *etc.*), exposure conditions (hazardous chemicals involved and workers’ exposure time, *etc.*), and exposure prevention strategies (engineering control measures, administrative control measures, and management measures) were collected and recorded by governmental industrial hygiene inspectors using a standardized questionnaire. All surveyors received appropriate training prior to conducting the field surveys.

The data obtained from the above survey program were used in the corrected EHI model. Chemical exposure risks (*i.e.*, EHI ratings) of various industries were identified, and the results were further used to prioritize the selected 25 manufacturing industries for initiating a national occupational exposure control program. Furthermore, factors affecting EHI ratings for industries of different priorities were determined, and possible prevention strategies were proposed.

## 3. Results and Discussion

### 3.1. Correcting the Proposed TI

**Figure 1:**
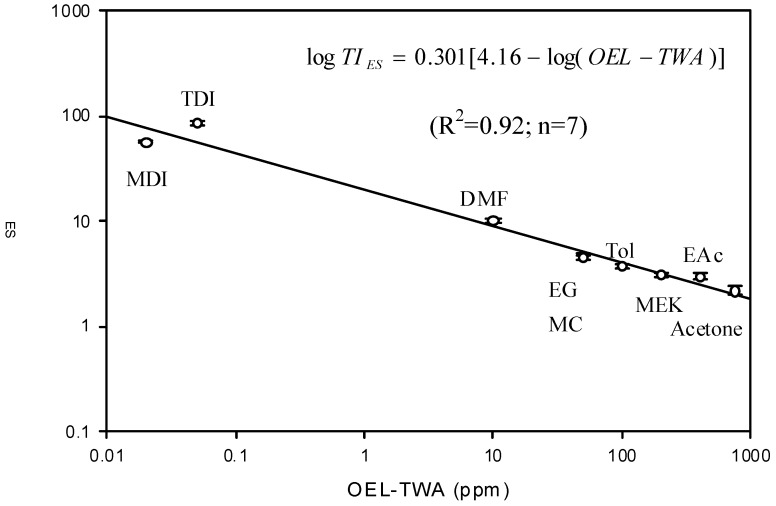
The relationship between TI_ES_ (mean ± 95% confidence interval) and the corresponding OEL-TWA.

Table 2 shows the ratings of TI obtained from the originally proposed ratings (TI_ori_) and the expert system (TI_E__S_). By reference to OEL-TWAs, both TI_ori_ and TI_E__S_ follow the same trend. Therefore, the results obtained from the expert system can be used as a basis for correcting the originally proposed ratings [24]. Figure 1 depicts the relationship between TI_E__S_ and their corresponding OEL-TWA. Clearly, TI_E__S_ were inversely proportional to the logarithm of OEL-TWA for nine chemicals found in seven selected enterprises. Considering tenfold increase in the magnitude of a chemical exposure would double its health effect, the following equation was used to address the relationship between TI_E__S_ and their corresponding OEL-TWA:

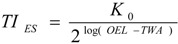
(3)

Assuming *K*_0_ = 2*^K^*, we converted the Equation (3) becomes the following:

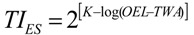
(4)

Based on the results obtained from the seven selected enterprises, this study yields the following equation:

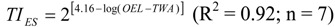
(5)

The above equation was used to correct TI_ori_ and the corrected values (TI_cor_) are shown in Table 2. By comparing TI_ori_ with TI_cor_; TI ratings are quite comparable for chemicals with high OEL-TWAs, but underestimations could be found for chemicals with low OEL-TWAs if the originally proposed ratings were adopted.

### 3.2. Correcting the Proposed EI

**Figure 2:**
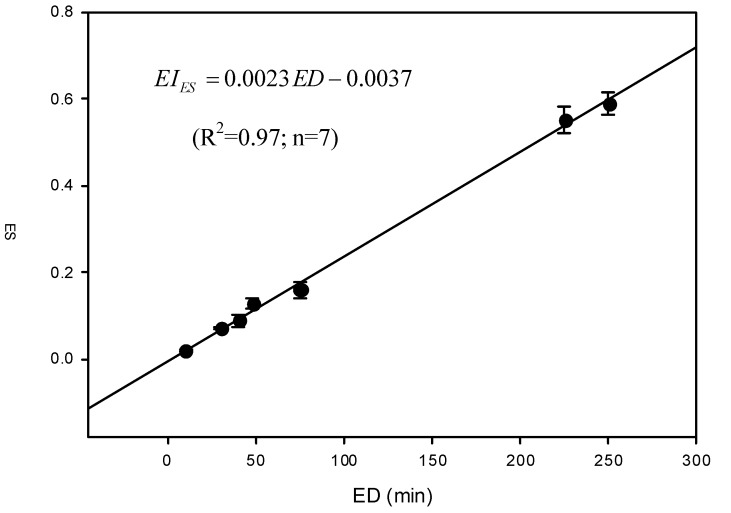
The relationship between EI_ES_ (mean ± 95% confidence interval) and the corresponding exposure duration (ED).

Table 2 also shows the ratings of EI obtained from original proposed ratings (EI_ori_) and the expert system (EI_ES_). In general, as the exposure durations (ED) increase, both EI_ori_ and EI_ES_ also increase. The above consistent trend suggests that the results obtained from the expert system can be used for correcting the originally proposed ratings. Figure 2 shows the relationship of EI_ES_ with their corresponding ED. The linear relationship suggests that a simple linear regression model would be adequate. Thus, this study yields a regression result as below:

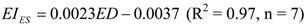
(6)

The above regression equation was used to correct EI_ori_ and the corrected values (EI_cor_) are shown in Table 2. Comparing EI_ori_ with EI_cor_, the former obviously is much less capable than the latter of discriminating those with low ED. The above result also suggests using category method for rating EI (*i.e.*, EI_ori_) would be inadequate from the aspect of practical application in the field.

### 3.3. Correcting the Proposed PDI

**Figure 3:**
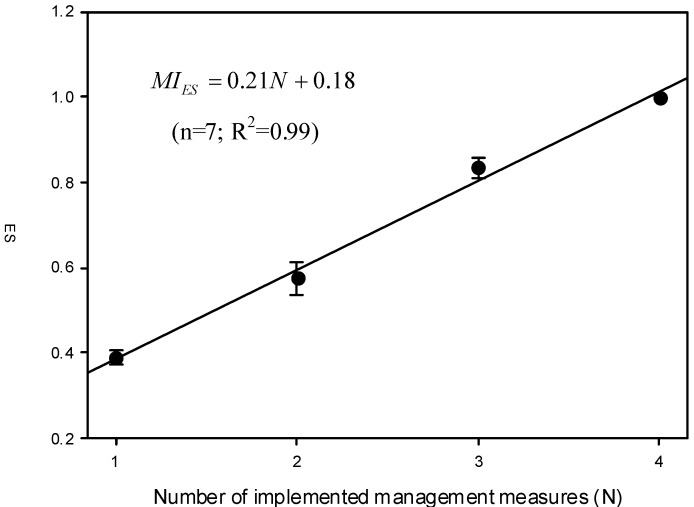
The relationship between MI_ES_ (mean ± 95% confidence interval) and the corresponding number of management measures implemented in the workplace (N).

The PDI index comprises two factors: the management index (MI) and protection index (PI). The rating of MI was based on the number of management measures (N) implemented in the workplace. As shown in Table 2, the ratings of MI for those obtained from the originally proposed ratings (MI_ori_) shares the same trend as that of the expert system (MI_ES_). Again, the above result suggests that the expert system can be used for correcting the originally proposed ratings. Figure 3 shows the relationship between MI_ES_ and the corresponding N. This study yields a regression result as follows:

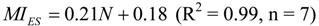
(7)

The above regression equation was used to correct MI_ori_ and the corrected values (MI_cor_) are also shown in Table 2. The similarity in both MI_ori_ and MI_cor_ suggests that both the originally proposed rating method and the expert system share similar judging philosophies. However, it should be noted that even when none of the four management measures are implemented in a workplace (*i.e.*, N = 0), the effectiveness of the management in the workplace is still not complete zero (*i.e.*, MI_cor_ ≠ 0). One reason for the above result might be that employers and the workers still have basic industrial hygiene knowledge, helping reduce workers’ exposures.

Table 2 also shows the ratings of PI obtained from originally proposed ratings (PI_ori_) and the expert system (PI_ES_). Again, the similarity in both PI_ori_ and PI_ES_ suggests that both rating systems share similar judging philosophies. Therefore, the expert system is used for correcting the originally proposed ratings in the present study. As shown in Table 2, the corrected PI ratings are 1.00, 0.74, 0.61, 0.42, 0.18, and 0.00 for EEe + PPEe, EEe + PPEn, EEp + PPEe, EEp + PPEn, EEn + PPEe and EEn + PPEn, respectively.

### 3.4. Comparing the Expert System Proposed EHI (EHI_ES_) with the Corrected EHI (EHI_cor_)

**Figure 4:**
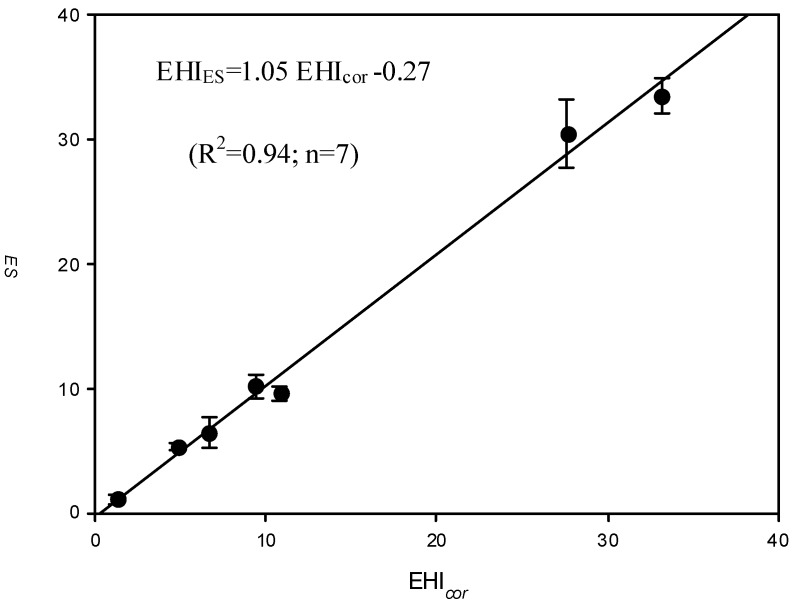
The relationship between EHI_ES_ (mean ± 95% confidence interval) and the corresponding corrected EHI (EHI_cor_).

In the present study, we assume the toxic effects of all involved chemicals are additive (*i.e.*, assuming no synergistic or antagonistic effects for a co-exposure). Therefore, the summation of EHI for each of all chemicals used in the enterprise was considered to be representative to the EHI of the enterprise. Figure 4 shows the relationship between the corrected EHI (EHI_cor_) and that obtained from expert system (EHI_ES_).

Through the linear regression, this study yields the following regression equation:

EHI_ES_ = 1.05 EHI_cor_ − 0.27 (R^2^ = 0.94; n = 7) (8)

Here, the regression coefficient (=1.05) and the intercept (=−0.27) are respectively approaching unity and zero, indicating that EHI_cor_ is truly derived from EHI_ES_. In particular, the high R^2 ^suggests that EHI_cor_ can be used to explain up to 94% of the variation in EHI_ES_. In the present study, the EHI_cor_ is adopted for predicting the occupational chemical exposure risk of various industries.

### 3.5. Implication of the Corrected EHI Model to a Nationwide Chemical Hazard Survey Database

In this study, the corrected EHI model (EHI_cor_) was used to access the occupational chemical exposure risks of twenty-five manufacturing industries based on the database obtained from a nationwide occupational chemical hazards survey program conducted from 2006 to 2009. In the present study, the grouping of industries is based on Standard Industrial Classification (SIC) Codes with 2 digits. The resultant EHI_cor_ were subsequently used to determine the control priorities of the twenty-five manufacturing industries. In principle, industries involved the use of chemicals with higher toxicity (*i.e.*, low OEL-TWA or high TI), longer exposure duration (*i.e.*, high EI), and less workers protection measures (*i.e*., high PDI resulting from a low MI (*i.e.*, less implemented management measures) and low PI (*i.e.*, implemented less effective control measures), would result in a higher EHI (*i.e.*, EHI = TI × EI × PDI).

Among the 25 studied manufacturing industries, those with higher EHI values would therefore have higher priorities when a national chemical exposure control strategy is proposed. In the present study, the distribution of EHI_cor_ for the 25 manufacturing industries studied was found with a mean and standard deviation as 14.7 and 12.0, respectively. The percentiles of EHI ratings were adopted for determining the control priorities among various industries. Here, the industries with the EHI_cor_ ≥ 90th percentile (*i.e.*, EHI_cor_ ≥ 32.4), 90th percentile > EHI_cor_ ≥ 70th percentile (*i.e.*, 32.4 > EHI_cor_ ≥ 20.6), 70th percentile > EHI_cor_ ≥ 50th percentile (*i.e.*, 20.3 > EHI_cor_ ≥ 10.4), and EHI_cor_ < 50th percentile (*i.e.*, EHI_cor_ < 10.4) were considered as first, second, third and fourth control priority industries, respectively. As shown in Table 3, in total 3, 5, 5, and 12 manufacturing industries were determined, respectively.

**Table 3:** The recommended four control priority manufacturing industries.

Control priority	Recommended industries	EHI_cor _*
**First priority industries (EHI_cor _ranking ≥ 90th percentile; EHI_cor_ ≥ 32.43)**		
	Plastic products manufacturing (*n* = 37)	43.47
	Petroleum products manufacturing (*n* = 7)	35.54
	Metal products manufacturing (*n* = 137)	33.17
**Second priority industries (90th percentile > EHI_cor _ranking ≥ 70th percentile; 32.43 > EHI_cor_ ≥ 20.61)**		
	Transportation manufacturing (*n* = 17)	31.32
	Electrical equipment manufacturing (*n* = 22)	27.23
	Electronic components manufacturing (*n* = 34)	23.46
	Chemical materials manufacturing (*n* = 94)	21.66
	Paper products manufacturing (*n* = 17)	21.44
**Third priority industries (70th percentile > EHI_cor _ranking ≥ 50th percentile; 20.61 > EHI_cor_ ≥ 10.41)**		
	Metalworking manufacturing (*n* = 49)	17.29
	Chemical products manufacturing (*n* = 16)	14.06
	Non-metallic mineral products manufacturing (*n* = 15)	12.58
	Leather products manufacturing (*n* = 9)	12.03
	Electronic products manufacturing (*n* = 30)	10.41
**Fourth priority industries (EHI_cor _ranking** **< 50th percentile; EHI_cor_** **< 10.41)**		
	Wood products manufacturing (*n* = 5)	10.16
	Printing and data storage products manufacturing (*n* = 20)	9.41
	Drug manufacturing (*n* = 5)	8.94
	Textile industrial (*n* = 15)	8.21
	Foods manufacturing (*n* = 50)	7.54
	Machinery and equipment manufacturing (*n* = 74)	5.71
	Furniture manufacturing (*n* = 10)	5.10
	Automotive manufacturing (*n* = 17)	2.48
**Fourth priority industries (EHI_cor _ranking** **< 50th percentile; EHI_cor_** **< 10.41)**		
	Machinery and equipment maintenance industry (*n* = 3)	2.28
	Rubber products manufacturing (*n* = 13)	1.33
	Beverage manufacturing (*n* = 3)	1.22
	Clothing products manufacturing (*n* = 3)	0.82

* The 95th percentile of the EHI_cor_.

**Figure 5:**
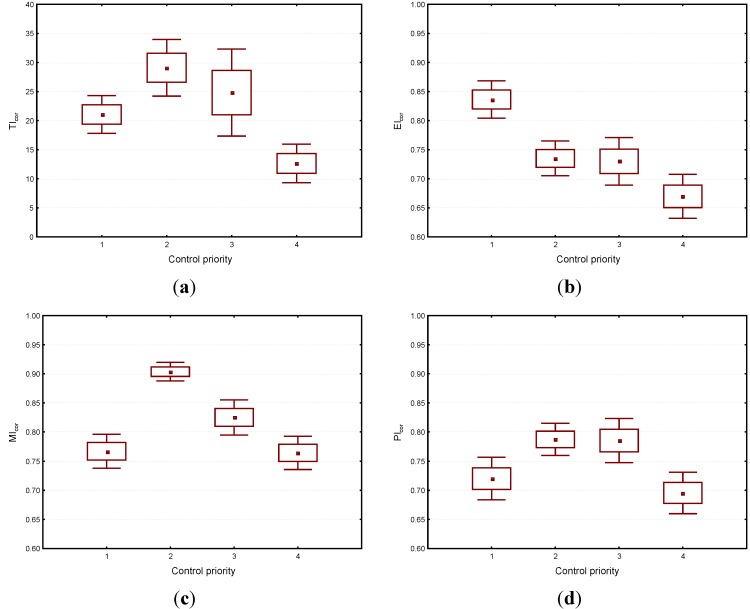
The predicted ratings for (**a**) toxicity index (TI), (**b**) exposure index (EI), (**c**) management index (MI), and (**d**) protection index (PI) for the four control priority industries using a whisker plots (the square dot inside the box is the mean, the bottom and top of the box are mean ± standard error (SE), and whiskers of the both ends are mean ± 1.96SE).

Figure 5 shows the predicted values of each individual index for the four control priority industries. For the first control priority industries, their high EHI values were clearly due to their high EI ratings and low ratings in both MI and PI. For reducing EI, it is suggested that both the rotation of workers and the automation of manufacturing process should be considered for reducing workers’ exposure durations. In addition to the above, governmental agencies should take measures, such as more intensive workplace inspection by governmental labor inspectors, to urge the industries to implement more management and effective control measures to reduce the occupational exposures of workers.

For the second and third control priority industries, their EHI ratings were obviously governed by their high TI values. Therefore, using less toxic chemicals to replace those currently used high toxicity chemicals might provide a solution for these two types of industries. For the fourth priority control industries, their low EHI ratings was mainly due to the involvement of relatively low toxic chemicals (*i.e.*, low TI ratings) and short exposure duration (*i.e.*, low EI ratings). However, it should be noted that their MI and PI ratings were relatively low among the four priority industries; as a result, implementation of more management and effective control measures are still required for the industries from the aspect of reducing the occupational exposure risks of workers.

## 4. Conclusions

Though the proposed EHI model is based on the concept of control banding, the proposed model involves the use of more sophisticated factors to reflect the toxicity and exposure potential of chemicals exposed to workers than those currently used models. Most importantly, the worker protection measures (such as the number of management measures, effectiveness of engineering control measures, and the use of personal protective equipment, *etc.*) were considered in the EHI model, providing more accuracy in predicting exposure intensities. Additionally, the proposed EHI model was further corrected by an expert system suggesting that it could be more effective in predicting the exposure risks of workers. The corrected EHI model is not only applicable to predicting chemical exposure risks for a single enterprise, but also applicable to determine exposure risks of various industries by using data in the databank obtained from a nationwide occupational chemical hazard survey. In this study, industries with four control priorities were identified by the resultant EHI ratings for initiating a national occupational exposure control program. In addition, factors affecting occupational chemical exposure risks were identified for industries with different priorities. The proposed model and its application to a national chemical exposure databank would be helpful for governmental agencies to develop suitable prevention strategies to reduce their occupational chemical exposure risks in industries. However, it should be noted that the TI were rated according to the OEL-TWA of a given chemical (including in both gas and particulate phases) designated for protecting workers from inhalatory exposures, making the proposed model inadequate for assessing workers’ dermal exposures. In addition, the model does not separate acute and chronic effects of the exposed chemicals, assumes the existence of synergistic effects for multiple chemical exposures, and carcinogens are treated in a simple way. Therefore, the proposed model should be used with caution. Finally, the proposed model can only be regarded as a semi-quantitative in nature, further validation through the comparison with quantitative exposure monitoring results is still needed in order to increase its predicting efficiency.

## References

[1] Marchant G., Bullock C., Carter C., Connelly R., Crane A., Fayerweather W., Johnson K. (2009). Applications and findings of an occupational exposure database for synthetic vitreous fibers. J. Occup. Environ. Hyg..

[2] Kauffer E., Vincent R. (2007). Occupational exposure to mineral fibers: Analysis of results stored on colchic database. Ann. Occup. Hyg..

[3] Yassin A., Yebesi F., Tingle R. (2005). Occupational exposure to crystalline silica dust in the United States, 1988–2003. Environ. Health Perspect..

[4] Marchant G., Amen M., Bullock C., Carter C., Johnson K., Reynolds J., Connelly F., Crane A. (2002). A synthetic vitreous fiber (SVF) occupational exposure database: Implementing the SVF health and safety partnership program. Appl. Occup. Environ. Hyg..

[5] National Institute for Occupational Safety and Health (NIOSH) (2009). Qualitative Risk Characterization and Management of Occupational Hazards: Control Banding (CB)—A Literature Review and Critical Analysis.

[6] Zalk D.M., Nelson D.I. (2008). History and Evolution of control banding: A review. J. Occup. Environ. Hyg..

[7] Money C.D. (2003). European experiences in the development of approaches for the successful control of workplace health risks. Ann. Occup. Hyg..

[8] Russell R.M., Maidment S.C., Brooke I., Topping M.D. (1998). An introduction to a UK scheme to help small firms control health risks from chemicals. Ann. Occup. Hyg..

[9] Oldershaw P. (2001). Control banding—A practical approach to judging control methods for chemicals. J. Prev. Med..

[10] Jackson H. (2002). Control banding—Practical tools for controlling exposure to chemicals. Asian-Pac. Newsl..

[11] Health and Safety Executive (HSE) (1999). The Technical Basis for COSHH Essentials: Easy Steps to Control Chemicals.

[12] Health and Safety Executive (HSE) (2003). COSHH Essentials: Easy Steps to Control Chemicals—Control of Substances Hazardous to Health Regulations HSG193.

[13] International Labour Organization (ILO) (2006). Draft ILO International Chemical Control Kit.

[14] Balsat A., de Graeve J., Mairiaux P. (2003). A structured strategy for assessing chemical risks, suitable for small- and medium-sized enterprises. Ann. Occup. Hyg..

[15] Money C., Bailey S., Smith M., Hay A., Hudspith B., Tolley D., Dobbie J., Jackson H. (2006). Evaluation of the utility and reliability of COSHH essentials. Letter to the Editor. Ann. Occup. Hyg..

[16] Paik S.Y., Zalk D.M., Swuste P. (2008). Application of a pilot control banding tool for risk level assessment and control of nanoparticle exposure. Ann. Occup. Hyg..

[17] Robichaud C.O., Tanzil D., Weilenmann U., Wiesner M.R. (2005). Relative risk analysis of several manufactured nanomaterials: An insurance industry context. Environ. Sci. Technol..

[18] Maidment S.C. (1998). Occupational Hygiene considerations in the development of a structured approach to select chemical control strategies. Ann. Occup. Hyg..

[19] Hashimoto H., Goto T., Nakachi N., Suzuki H., Takebayashi T., Kajiki S., Mori K. (2007). Evaluation of the control banding method—Comparison with measurement-based comprehensive risk assessment. J. Occup. Health.

[20] Jones R.M., Nicas M. (2006). Margins of safety provided by COSHH Essentials and the ILO Chemical Control Toolkit. Ann. Occup. Hyg..

[21] Tischer M., Bredendiek-Kämper R., Poppek U. (2003). Evaluation of the HSE COSHH Essentials exposure predictive model on the basis of BAuA field studies and existing substances exposure data. Ann. Occup. Hyg..

[22] Finny D.J. (1971). Quantal Responses and the Dose-response Curve. Probit Analysis.

[23] Finny D.J. (1971). Estimation of the Median Effective Dose-response Curve. Probit Analysis.

[24] Tait K. (1992). The workplace exposure assessment expert system (WORKSPERT). Am. Ind. Hyg. Assoc. J..

[25] Fritschi L., Nadon L., Benke G., Lakhani R., Latreille B., Parent M.E., Siemiatycki J. (2003). Validation of expert assessment of occupational exposures. Am. J. Ind. Med..

